# *Marshallagia marshalli* Antigen Strengthens Dendritic Cell Mediated T Lymphocyte Regulation on Asthmatic Patients

**Published:** 2020

**Authors:** Fatemeh HEMATI, Majid MIRSADRAEI, Milad HEMATI, Hadi MOHEBALIAN, Hassan BORJI

**Affiliations:** 1. Department of Pathobiology, Faculty of Veterinary Medicine, Ferdowsi University of Mashhad, Mashhad, Iran; 2. Department of Clinical Sciences, Mashhad Azad University of Medical Sciences, Mashhad, Iran; 3. Atherosclerosis Prevention Research Center, Mashhad University of Medical Sciences, Mashhad, Iran

**Keywords:** *Marshallagia marshalli*, Dendritic cells, T-Lymphocytes, Asthma

## Abstract

**Background:**

The current study was conducted to investigate the antigenic effect of *Marshallagia marshalli* on the treatment of asthma by measuring the secreted inhibitory cytokine.

**Methods:**

Case patients and controls were selected from clinics in Mashhad, Khorasan Razavi Province, Northeastern Iran in 2017–18. In this experimental study, peripheral blood mononuclear cells (PBMCs) were isolated from 15 patients with asthma and 10 healthy controls and were cultured. PBMCs were then converted to tolerogenic DCs through exposure to GM-CSF, IL-4 and *M. marshalli* antigen. Then, tolerogenic DCs were exposed to autologous T cells for five days and finally, the level of secreted TGF-β1 was measured.

**Results:**

The mean TGF-β1 level in the control and control groups was 210.2 ± 8.2 and 225.4 ± 6.1 pq/ml, respectively. The results showed that TGF-β1 levels in both groups significantly increased in both groups (*P*<0.001). In addition, TGF-β1 levels in the case group were significantly higher than the control group (*P*<0.001).

**Conclusion:**

*M. marshalli* antigen increase the level of TGF-β1 and can create antigen-bearing dendritic cells and shift T lymphocytes to the regulatory type. This parasite can be used in dendritic cell therapy to control allergic diseases.

## Introduction

Asthma is a complex and chronic inflammatory disease that affects more than 300 million people worldwide, and the number of infected people is increasing (1–3). This disease is accompanied by pulmonary inflammation, increased mucus secretion, reversible airway obstruction and wheezing (4, 5). Despite the considerable effect of current treatments, including corticosteroids and β2 agonists in most patients, there is no definitive treatment for this condition (4–6). In addition, the administration of systemic corticosteroids causes many side effects in patients who are resistant to the treatment (7, 8). Therefore, there is always a need for new treatments.

The inflammation caused by asthma is originated from Th2 lymphocytes, which secrete a group of cytokines including IL-5, IL-4, IL-3, IL-13 and GM-CSF (2, 9, 10). Th2 cytokines are responsible for the regular management of an allergic inflammatory cascade that occurs in asthma, such as changing the class of secretory antibodies from plasma cells to IgE (IL-13 and IL-4), the differentiation of mast cells (IL-3 and IL-4 and IL-13), maturation and survival of eosinophils (IL-3, IL-5 and GM-CSF) and summons of basophils (IL-3 and GMCSF) (11–13).

The prevalence of asthma in developed countries is higher than in developing countries, which, based on the hygiene hypothesis, is due to the reduction of contamination with bacteria and worm, and high standards of health in developed countries (14–16).

Worms have infected about one-fourth of the world's population and rarely cause death. There have been many advances in perceiving the host-parasite relationship and the involved cellular and molecular mechanisms. The mechanisms include the induction of IL-10 and TGF β regulatory cytokines and the summons of DC4+CD25+FOXP3 T cell (T-reg) and the activity of anti-inflammatory alternative macrophage phenotype (17, 18). Many studies have been conducted regarding the positive effects of different types of worms on controlling allergic diseases proven the hygiene hypothesis. In developing countries, contamination with worm parasites has been associated with a reduction in the incidence of allergies, asthma and autoimmune diseases (19, 20).

Nematode parasites such as *Marshallagia marshalli* may increase their survival by shifting immune responses to regulatory immunity (18, 21). For this reason, chronic worm infections may protect the host against allergic diseases due to extensive immunosuppression. This extensive immunosuppression can generally lead to a decrease in T cell responsiveness through the activity of T-reg cells and by regulating the effects of immune cells such as macrophages, dendritic cells, and topical stromal cells (21, 22). According to the results of research on different types of worms and the observation of regulatory effects in their inflammatory responses, a hypothesis arises that states dendritic cells and T lymphocytes can shift to dendritic toluene cells and regulatory T cell using worm antigens (23, 24). This process is effective in treating autoimmune and inflammatory diseases. For this reason, dendritic cell therapy has been used to treat many diseases, such as cancers in recent years (25–28). Dendritic cells are the only cells that can activate the T lymphocyte as the antigen-presenting cells, and shift the T lymphocytes into helper T lymphocytes (25, 27). Nowadays, DCs are used to produce vaccines for the treatment of many diseases (29, 30). However, the hypothesis that states parasitic antigens can be used to treat allergic diseases has not been definitively proved yet.

In this study, tolerogenic dendritic cells and regulatory T lymphocytes were produced using *M. marshalli* antigens and we attempted to investigate the antigenic effect of this parasite on the treatment of asthma by measuring the secreted inhibitory cytokine.

## Materials and Methods

Case patients and controls were selected from clinics in Mashhad in Khorasan Razavi Province in Northeastern Iran in 2017–18. In this experimental study, 25 samples including 15 patients with asthma as case group and 10 healthy subjects as control group were randomly included in the study. The selection of samples in the case group was confirmed through examination by asthma and allergy specialist. The inclusion criteria were suffering from various underlying diseases such as autoimmune diseases, immunodeficiency, genetic problems, malignancy, and viral diseases. Then, 5 ml peripheral blood was collected from each sample and after isolating the PBMCs using Falcon, the monocyte cells were cultured in a 25 ml flask.

A written informed consent was obtained from each patient before entering the study. The human investigation committee at Medical University of Mashhad approved the study protocol.

### Preparation of somatic *M. marshalli* antigens

First, a large number of contaminated rennet were transferred from Industrial slaughterhouse of Mashhad to the Parasitology Laboratory of the Faculty of Veterinary Medicine of Ferdowsi University of Mashhad. The contents of the rennet were cleared and the contents were poured into a plate containing PBS. Subsequently, the mature male *M. marshalli* parasites were identified and isolated based on their morphology using loop device.

They were then washed with sterile PBS solution during 4 steps. Then, the worms were fragmented inside a sterile petri dish using scalpel and were transferred into sterile microtubules. It was then homogenized several times with homogenizer W130 for 20 sec each time in the vicinity of ice. The homogenized product was centrifuged at 1500 rpm at 4 °C for 5 min. The supernatant was removed and the sediment was discarded.

### Production of mature dendritic cells

After culture, monocyte cells converted to dendritic cells by adding IL-4 and GM-CSF cytokines in a 3-day process. After the initial culture of PBMC cells, the cells were passaged in a new flask with sterile RPMI+10% FBS medium and placed in a CO2 incubator for two hours. The flask was removed from the incubator and the supernatant was discarded, and 10 μl GM-CSF and 10μl IL-4 were added. On the third day, 10 μl GM-CSF and IL-4 were added to the cells again.

To convert the dendritic cells to tolerogenic type, the following protocol was implemented: On the fourth day, 15 μL of the somatic *M. marshalli* parasite with a concentration of 289.91 μg/ml was added to the flask of dendritic cells. After 24 h of exposure to *M. marshalli* parasite antigens, 10 μl of LPS was added to the flask (5). Eventually, after two days of exposure, the maturity of the dendritic cells was confirmed by observing pseudopods, and the flask contents containing mature dendritic cells were transferred to sterile Falcon.

### Production of autologous T lymphocytes

A portion of the PBMC cells were separated from the primary culture, were transferred to a new flask with sterile RPMI+10% FBS medium and were placed in an CO2 incubator for two hours. After two hours, the flask was removed from the incubator and its contents (T-lymphocytes) entered the sterilized Falcon.

### Quantitative measurement of TGF-β1 cytokine

Mature dendritic cells and T lymphocytes with ratios of 1:10 and 1:5 were transferred to sterile microplate with sterile RPMI+10% FBS medium and cultured for five days. After 5 d of proximity of dendritic cells with lymphocytes, the contents of wells were transferred to microtubule (31). Then, they were centrifuged at 1500 rpm for 10 min and the supernatant was removed and the contents were stored in freezer at −80 °C until TGF-β1 cytokine was measured. Finally, the quantitative measurement of TGF-β1 cytokine was done by ELISA test (Human TGF beta 1 ELISA Kit) according to the procedure determined by the manufacturer. The test sensitivity in this kit was 2 pq/ml.

### Data Analysis

The results were analyzed using SPSS 16 software (Chicago, IL, USA). Descriptive data were composed of the frequency distribution table, central indexes, distribution and percentages. Continuous quantitative data were compared between the cases and control groups using the independent T-test and Chi-Square test. A *P*<0.05 was considered as the level of significance.

## Results

Twenty-five samples were included in this study, including 15 patients with asthma as case group and 10 healthy subjects as control group. The case group consisted of 8 men (53.3%) and 7 women (46.7%), and the control group consisted of 5 men (50%) and 5 women (50%). The mean age of these samples in the case and control groups was 44.87±9.38 and 39.1±10.86 yr, respectively. There was no significant difference between the two groups in terms of age and gender (*P*>0.05) (Table 1).

**Table 1:** Demographics and clinical characteristics of cases and controls

***Variable***	***Cases n (%)***	***Controls n (%)***	**P*-value***
Gender			
Male	8 (53.3%)	5 (50%)	0.089
Female	7 (46.7%)	5 (50%)	
Age			
Age (yr)	44.87±9.38	39.1±10.86	0.061
Range	20–55	20–50	-
Family history of disease			*P*-value
Parents	8 (53.3%)	-	<0.05
Siblings	7 (46.6%)	-	
2nd and 3rd generation	13 (86.6%)	-	
Clinical symptoms			
Shortness of breath	11 (73.33%)	-	<0.05
Cough	15 (100%)	-	
Wheezing	12 (80%)	-	
Chest Tightness	10 (66.7%)	-	
Feelings Of Suffocation	9 (60%)	-	
Nasal congestion	9 (60%)	-	
Rhinorrhea	11 (73.33%)	-	
Total	15	10	-

In examining the clinical symptoms of samples with asthma, all samples suffered from shortness of breath, and coughing. Clinical symptoms in 80% of cases were wheezing, 66.7% had chest tightness and 60% had feelings of suffocation. Furthermore, 53.3% of the samples stated that they had productive coughs with mucus. In addition to asthma, some samples in the case group suffered from other allergic diseases such as allergic rhinitis (66.6%), eczema (20%), hives (26.6%), skin allergies (26.6%), food allergies (33.3%), and drug allergies (13.3%) (Table 1). After preparing mature dendritic cells that became tolerogenic by the somatic antigens of *M. marshalli* parasite and its proximity to the autologous T cells, they were exposed to these cells for 5 d and were examined for stimulation of T cells.

**Fig. 1:**
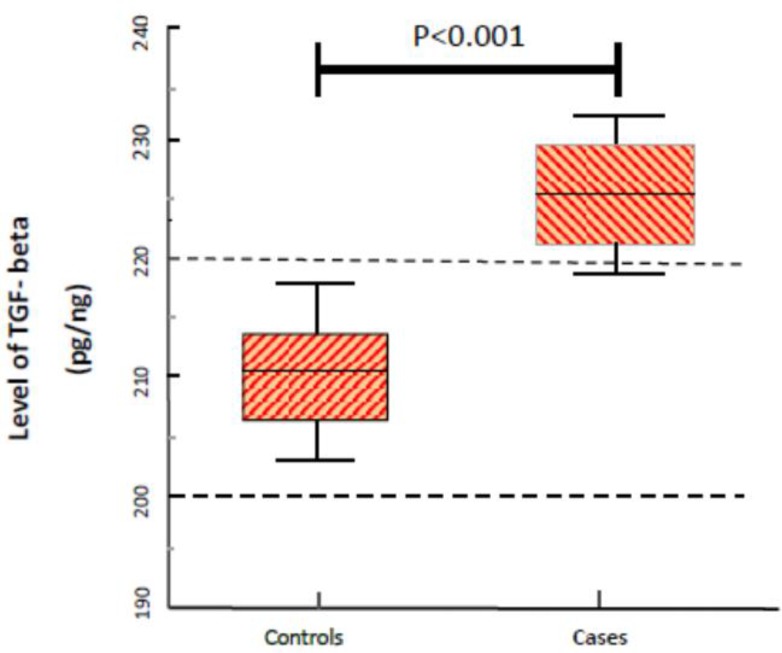
The Mean±SD of TGF-β1 in the samples analyzed by case and control group

TGF-β1 cytokine was measured to examine the stimulation of T cells by tolerogenic dendritic cells. Mean TGF-β1 level in the case and control groups was 210.2 ± 8.2 and 225.4 ± 6.1 pq/ml, respectively. The results of TGF-β1 level data analysis in the samples of case and control groups showed that TGF-β1 levels in the case group were significantly higher than the control group (*P*<0.001) (Fig. 1).

There was no significant relationship between gender and age of samples and TGF-β1 level in both case and control groups. In addition, there was no significant relationship between family history of asthma and TGF-β1 levels. Moreover, there was no significant relationship between the clinical symptoms of the samples in the case group and TGF-β1 levels. However, there was a significant relationship between TGF-β1 levels in patients with history of comorbidities and allergic rhinitis (*P*=0.006), skin allergy (*P*=0.028), food allergy (*P*=0.004) and eczema (*P*=0.034).

In the case group, TGF-β1 levels in samples with a history of comorbidities and asthma was higher than other samples (Table 2).

**Table 2:** The relationship between TGF-β1 levels in patients with history of comorbidities by Asthma

***The history of comorbidities by Asthma***		***N (%)***	***TGF-β1 levels (pq/ml)***	**P*-value***
Eczema	Yes	3 (20)	230.43±0.7	0.034
	No	12 (80)	224.12±3.91	
Eczema				
Allergic Rhinitis	Yes	10 (66.6)	227.91±2.8	0.006
	No	5 (33.4)	220.34±0.98	
Hives	Yes	4 (26.6)	226.52±5.22	0.656
	No	11 (73.4)	224.97±4.2	
Skin Allergy	Yes	4 (26.6)	230.5±1.04	0.028
	No	11 (73.4)	223.52±3.45	
Food Allergy	Yes	5 (33.4)	230.36±0.95	0.004
	No	10 (66.6)	222.9±2.91	
Drug Allergy	Yes	2 (13.3)	223.0±3.95	0.544
	No	13 (86.7)	225.75±4.44	

## Discussion

In recent years, the incidence of various types of allergic diseases has increased in many countries (3–5). Asthma is one of the most important allergic diseases. The characteristics of this disease are excessive production of cytokines secreted from Th2 cells, such as Interleukin 4, 5, and 13, and the accumulation of eosinophils in the submucosal intestinal layers (32). By summoning other cells, these cells cause mucosal hyperemia and cause the symptoms of the disease (1, 32).

Dendritic cell therapy, also called dendritic vaccine, is a new immunotherapy capability used to treat diseases like cancer. Today, the idea of using dendritic drugs for the treatment of asthma and allergic diseases has also been raised, and parasitic worm antigens are used for converting dendritic cells into T-cells (25, 27, 29, 30).

Nematode parasites such as *M. marshalli* may increase their survival by shifting immune responses to regulatory immunity (18, 21). For example, they interfere with the balance in the Th1/Th2 response and lead to the production of a large number of regulatory cytokines such as IL-10 and TGF-β, as well as imitation of host proteins that convert immune response to immune tolerance (33, 34). Therefore, chronic worm infections may protect the host against allergic diseases due to extensive immunosuppression. This extensive suppression, in general, can lead to a decrease in T cell responsiveness by the activity of T-reg and by regulating the effects of immune cells such as macrophages, dendritic cells, and stromal cells, which leads to the creation of a non-inflammatory environment with high levels of IL-10 and TGF-β (11–13, 22). These cytokines generally result in the inhibition of T and B cell responses.

The results of this study showed that TGF-β1 level increased in both case and control groups due to exposure to somatic *M. marshalli* antigens with lymphocytes. The *M. marshalli* somatic antigens can shift lymphocyte cells in healthy people and patients to regulatory lymphocyte and provide a basis for treatment and control of asthma. Moreover, history of comorbidities by Asthma with other allergic diseases could significantly increase TGF-β1 levels.

Similar studies confirm the results of our study. The effect of ES *M. marshalli* antigens on peripheral blood mononuclear cells (PBMCs) of 25 healthy samples and 25 samples with asthma was investigated. The expression of TGF-β as a gene secreted from T-reg in co-culture with ES *M. marshalli* antigen increased significantly in healthy samples and samples with asthma (18). In addition, in an in vivo study, the effect of ES and somatic *M. marshalli* was examined by investigating the inflammatory cell infiltration into the bronchoalveolar lavage fluid (BALF), pathological changes and IgE response, and it was determined that the pathological changes decreased in the study samples. Furthermore, the levels of inhibitory TGF-β cytokine increased in the tissue homogeneity, serum, and bronchoalveolar lavage fluid, with a significant increase in the homogeneity of lung tissue (35).

In addition, many studies were conducted on the effects of other parasite antigens on the immune system. Jin et al. examined murine dendritic cell line DC2.4 and immature dendritic cells of mouse bone marrow with the antigen of *Clonorchis sinensis* parasite and without this antigen. The rate of TGF-β cytokine production increased significantly (36).

In a study, the effect of *Schistisoma mansoni* antigen was examined on PBMC. TGF-β levels increased in the examined cells (37). *S. japonicum* antigen was effective in decreasing CD4 + CD25 + T cell production and increasing T-reg in mice. The use of *Schistosoma* parasite increases the level of IL-10 and decreases the expression of Th2 cytokines and inhibits the progression of asthma (38).

The effect of murine filariasis *(Litomosoides sigmodontis)* was examined on the mouse model of asthma, and the results showed that infection with this parasite significantly reduced the phenotypic aspects of asthma, including eosinophilia and Th2 cytokine secretion. Collecting and culturing splenic mononuclear cells determined that TGF-β levels increased significantly (39).

## Conclusion

The proximity of *M. marshalli* worm antigens to DC cells could increase TGF-β1 levels in the study samples in both case and control groups. Furthermore, TGF-β1 levels in the case group were significantly higher than the control group. Parasitic antigens can create tolerogenic dendritic cells and shift the T lymphocytes to the regulatory type. Therefore, these results can provide the basis for future asthma treatments.

## References

[1] Lambrecht BN Hammad H The immunology of asthma. Nature Immunol. 2015;16(1):45. 25521684 10.1038/ni.3049

[2] Pelaia G Vatrella A Busceti MT Cellular mechanisms underlying eosinophilic and neutrophilic airway inflammation in asthma. Mediators Inflamm. 2015;2015:879783. 25878402 10.1155/2015/879783 PMC4386709

[3] Rico-Rosillo G Vega-Robledo GB Silva-Garcia R [Epigenetics, environment and asthma]. Revista alergia Mexico (Tecamachalco, Puebla, Mexico : 1993). 2014;61(2):99–109. 24915622

[4] Bergmann KC Asthma. Chem Immunol Allergy. 2014;100:69–80. 24925386 10.1159/000358575

[5] Cavkaytar O Sekerel BE Baseline management of asthma control. Allergol Immunopathol (Madr). 2014;42(2):162–8. 23265257 10.1016/j.aller.2012.10.004

[6] Chung KF Targeting the interleukin pathway in the treatment of asthma. Lancet. 2015;386(9998):1086–96. 26383000 10.1016/S0140-6736(15)00157-9

[7] Kourie HR Klastersky J Immune checkpoint inhibitors side effects and management. Immunotherapy. 2016;8(7):799–807. 27349979 10.2217/imt-2016-0029

[8] Fahy JV Type 2 inflammation in asthma present in most, absent in many. Nat Rev Immunol.. 2015;15(1):57. 25534623 10.1038/nri3786 PMC4390063

[9] Hondowicz BD An D Schenkel JM Interleukin-2-dependent allergen-specific tissue-resident memory cells drive asthma. Immunity. 2016;44(1):155–66. 26750312 10.1016/j.immuni.2015.11.004 PMC4720536

[10] Capone M Maggi L Santarlasci V Chitinase 3-like-1 is produced by human Th17 cells and correlates with the level of inflammation in juvenile idiopathic arthritis patients. Clin Mol Allergy. 2016;14(1):16. 27826220 10.1186/s12948-016-0053-0 PMC5100333

[11] Holgate ST Innate and adaptive immune responses in asthma. Nat Med. 2012;18(5):673. 22561831 10.1038/nm.2731

[12] Ramadani F Upton N Hobson P Intrinsic properties of germinal center-derived B cells promote their enhanced class switching to IgE. Allergy. 2015;70(10):1269–77. 26109279 10.1111/all.12679 PMC4744720

[13] Tellier J Shi W Minnich M Blimp-1 controls plasma cell function through the regulation of immunoglobulin secretion and the unfolded protein response. Nat immunol. 2016;17(3):323. 26779600 10.1038/ni.3348 PMC4757736

[14] Legaki E Gazouli M Influence of environmental factors in the development of inflammatory bowel diseases. World J Gastrointest Pharmacol Ther. 2016;7(1):112–25. 26855817 10.4292/wjgpt.v7.i1.112 PMC4734944

[15] Boughattas I Hattab S Boussetta H Impact of heavy metal contamination on oxidative stress of *Eisenia andrei* and bacterial community structure in Tunisian mine soil. Environ Sci Pollut Res Int. 2017;24(22):18083–95. 28624946 10.1007/s11356-017-9449-8

[16] Maltman C Donald LJ Yurkov V Two distinct periplasmic enzymes are responsible for tellurite/tellurate and selenite reduction by strain ER-Te-48 associated with the deep sea hydrothermal vent tube worms at the Juan de Fuca Ridge black smokers. Archives Microbiol. 2017;199(8):1113–20. 10.1007/s00203-017-1382-1 28432382

[17] Santos LN Pacheco LGC Pinheiro CS Recombinant proteins of helminths with immunoregulatory properties and their possible therapeutic use. Acta tropica. 2017;166:202–11. 27871775 10.1016/j.actatropica.2016.11.016

[18] Jabbari Azad F Kiaee F Rezaei A Downregulation of immune responses in asthmatic humans by ES products of *Marshallagia marshalli*. Clin Respir J. 2017;11(1):83–89. 25919312 10.1111/crj.12309

[19] Finlay CM Walsh KP Mills KH Induction of regulatory cells by helminth parasites: exploitation for the treatment of inflammatory diseases. Immunol Rev. 2014;259(1):206–30. 24712468 10.1111/imr.12164

[20] van Riet E Hartgers FC Yazdanbakhsh M Chronic helminth infections induce immunomodulation: consequences and mechanisms. Immunobiology. 2007;212(6):475–90. 17544832 10.1016/j.imbio.2007.03.009

[21] Shirvan SP Ebrahimby A Dousty A Somatic extracts of *Marshallagia marshalli* downregulate the Th2 associated immune responses in ovalbumin-induced airway inflammation in BALB/c mice. Parasites & Vectors. 2017;10(1):233. 28494800 10.1186/s13071-017-2159-8 PMC5427607

[22] Akdis M Immune tolerance in allergy. Curr Opin Immunol. 2009;21(6):700–7. 19700272 10.1016/j.coi.2009.07.012

[23] Sojka DK Huang YH Fowell DJ Mechanisms of regulatory T-cell suppression–a diverse arsenal for a moving target. Immunology. 2008;124(1):13–22. 18346152 10.1111/j.1365-2567.2008.02813.x PMC2434375

[24] Beyer M Schultze JL Regulatory T cells: major players in the tumor microenvironment. Curr Pharm Des. 2009;15(16):1879–92. 19519430 10.2174/138161209788453211

[25] Takahashi T Dendritic cell therapy. Nihon Rinsho. 2001;59(12):2421–6. 11766350

[26] De Vleeschouwer S Van Calenbergh F Demaerel P Transient local response and persistent tumor control in a child with recurrent malignant glioma: treatment with combination therapy including dendritic cell therapy: case report. J Neurosurg. 2004;100(5 Suppl Pediatrics):492–7. 15287461 10.3171/ped.2004.100.5.0492

[27] Hilkens C Isaacs J Tolerogenic dendritic cell therapy for rheumatoid arthritis: where are we now? Clin Exp Immunol. 2013;172(2):148–57. 23574312 10.1111/cei.12038 PMC3628318

[28] McCurry KR Colvin BL Zahorchak AF Regulatory dendritic cell therapy in organ transplantation. Transpl Int. 2006;19(7):525–38. 16764631 10.1111/j.1432-2277.2006.00306.x

[29] Banchereau J Palucka AK Dendritic cells as therapeutic vaccines against cancer. Nat Rev Immunol. 2005;5(4):296. 15803149 10.1038/nri1592

[30] Palucka K Banchereau J Dendritic-cell-based therapeutic cancer vaccines. Immunity. 2013;39(1):38–48. 23890062 10.1016/j.immuni.2013.07.004 PMC3788678

[31] Inaba K Metlay JP Crowley MT Dendritic cells pulsed with protein antigens in vitro can prime antigen-specific, MHC-restricted T cells in situ. J Exp Med. 1990;172(2):631–40. 2373994 10.1084/jem.172.2.631 PMC2188342

[32] Lloyd CM Hessel EM Functions of T cells in asthma: more than just T H 2 cells. Nat Rev Immunol. 2010;10(12):838. 21060320 10.1038/nri2870 PMC3807783

[33] Hagel I Cabrera M Di Prisco MC Helminthic Infections and Asthma: Still a Challenge for Developing Countries. Respiratory Disease and Infection-A New Insight: InTech; 2013.

[34] Moradpour N Borji H Razmi G Pathophysiology of *Marshallagia marshalli* in experimentally infected lambs. Parasitology. 2013;140(14):1762–7. 24007639 10.1017/S0031182013001042

[35] Shirvan SP Borji H Movassaghi A Anti-inflammatory potentials of excretory/secretory (ES) and somatic products of *Marshallagia marshalli* on allergic airway inflammation in BALB/c mice. Iran J Parasitol. 2016;11(4):515. 28127363 PMC5251180

[36] Jin Y Wi HJ Choi M-H Regulation of anti-inflammatory cytokines IL-10 and TGF-β in mouse dendritic cells through treatment with Clonorchis sinensis crude antigen. Exp Mol Med. 2014;46(1):e74. 24480801 10.1038/emm.2013.144 PMC3909892

[37] Remoue F To Van D Schacht AM Gender-dependent specific immune response during chronic human Schistosomiasis haematobia. Clin Exp Immunol. 2001;124(1):62–8. 11359443 10.1046/j.1365-2249.2001.01495.x PMC1906031

[38] Yang J Zhao J Yang Y *Schistosoma* *japonicum* egg antigens stimulate CD4+ CD25+ T cells and modulate airway inflammation in a murine model of asthma. Immunol. 2007;120(1):8–18. 10.1111/j.1365-2567.2006.02472.x PMC1890919 17042799

[39] Dittrich AM Erbacher A Specht S Helminth infection with Litomosoides sigmodontis induces regulatory T cells and inhibits allergic sensitization, airway inflammation, and hyperreactivity in a murine asthma model. J Immunol. 2008;180(3):1792–9. 18209076 10.4049/jimmunol.180.3.1792

